# Early introduction of the multi-disciplinary team through student Schwartz Rounds: a mixed methodology study

**DOI:** 10.1186/s12909-022-03549-7

**Published:** 2022-07-03

**Authors:** Harry Abnett, Robert Tuckwell, Lucy Evans

**Affiliations:** Medical Education Department, Princess Alexandra Hospital (PAH), Hamstel Road, Harlow, CM20 1QX UK

**Keywords:** Schwartz Rounds, Reflective Practice, Multi-disciplinary team, Undergraduate, Teaching, Group Reflection

## Abstract

**Background:**

Medical education has changed continually throughout the covid-19 pandemic, creating additional stress for medical students. Personal reflection can empower an individual to adapt to new challenges, and reflection has gradually become incorporated into medical student training. Schwartz Rounds (SR) offer a compassionate group reflective forum for healthcare staff. SRs have been extensively introduced throughout the NHS, however medical student rounds are yet to be widely adopted. Entirely unresearched is how the multi-disciplinary team impacts a medical student SR.

This study aims to compare medical student experience of a single-discipline and a multi-discipline SR using mixed methodology.

**Methods:**

Two virtual SRs were run at an NHS district general hospital, using the existing structure of the Trust’s rounds. The first round included only medical students on placement at the hospital, whereas the second round also involved other student health disciplines. Following each round Likert scale questionnaires were collected, and focus groups were held with a small number of participants. Quantitative analysis used median averages as well direct comparison of scores for each round. Qualitative data from the focus groups underwent thematic analysis.

**Results:**

The quantitative data showed a positive response to both styles of student SRs, with over 87% of participants at both rounds stating they intended to attend further rounds. Direct comparison between the two rounds showed higher feedback scores for the single-discipline round. Qualitative analysis showed strong student interest in further group reflection, noting the value of SRs in improving workplace culture and inter-professional relationships. The analysis also highlighted frustrations with the existing SR structure, namely large group sizes and scripted panellists.

**Conclusions:**

Both data sets showed a strong positive response to SRs, and a desire to attend again. There is some evidence to suggest the addition of multiple student disciplines at SRs impaired medical student reflection. Changes to the format of the round could result in even greater success in student rounds.

## Background

### Mental health in the NHS

Before the arrival of Covid-19 the National Health Service (NHS) had been facing an epidemic within its own staff. Staff absences rates in the NHS are higher than the general economy, and for the last decade mental health illness has been the single most common reason for staff absence in the NHS [1]. As sickness rates increased during the pandemic the single biggest cause of absence remained psychiatric illness, despite escalating rates of infections [2]. The pandemic not only increased work stress but also removed many of the coping mechanisms people rely on, like social interactions with family and friends [3]. Whilst quantitative data on medical students mental health is harder to find, they nonetheless represent a high risk demographic. There is reluctance from students to seek formal mental health support, since they worry the perceived stigma around seeking help could impact upon their fitness to practice [4].

### Schwartz rounds

It is no coincidence that as national focus increases on mental health and burnout in the NHS, so too has focus increased on strategies to alleviate these problems [5]. Schwartz Rounds (SR) are one example of these mitigating strategies. They were created by Ken Schwartz, an American health attorney who was diagnosed with terminal lung cancer in 1994. In the following years, Schwartz found that “*what mattered to him most as a patient were the simple acts of kindness from his caregivers, which he said made “the unbearable bearable".”* [6]. As part of his legacy, The Schwartz Centre was established, a Boston based charity with the mission ‘to *put compassion at the heart of healthcare through programs, education and advocacy.’* [7]. SRs function as a forum for healthcare staff to gather and listen to personal reflections from colleagues. Attendees are then encouraged to contribute their own reflections in response. Not all attendees are expected to verbally contribute during the round, but it is hoped the discussions trigger each attendee to internally reflect during or after the event. This method of reflection can be traced back to Frederik Schon’s 1991 model, which states reflection occurs both in and on action, that is both during and after the event [8]. The ultimate aim of the rounds is threefold: reduce staff stress, improve understanding of healthcare challenges and ultimately empower staff to provide truly compassionate care [9]. Rounds are designed to “not focus on the clinical aspects of patient care” and are thus not a clinical exercise. As such they do not involve significant medical details other than to provide context. Patients are not identified or involved in the rounds, although SRs exist ultimately to improve patient experience [10]. After a successful UK pilot in 2009 [11], the rounds have been increasingly used as a method of reflective practice across the NHS. Over 130 NHS trusts actively run SRs, as well as 35 hospices and eight universities [6]. Rounds are open to all clinical and non-clinical staff, including students, to ensure the full multi-disciplinary team (MDT) is engaged. Licensing of rounds and facilitator training is managed by the Point of Care Foundation (POCF), a UK charity that is partnered with The Schwartz Centre [12]. The POCF provides guidance on preparing and running rounds, including example topics.

### Student Schwartz rounds

The General Medical Council (GMC) acknowledges that a range of reflective mediums is vital to students’ success and growth as a reflective practitioner, but medical schools must balance this against their own need to formally monitor student development [13]. Whilst they can be a challenge to implement, a National Institute for Health and Research (NIHR) prospective study showed that SR’s provide a unique form of support for staff, and result in a significant improvement in psychological wellbeing [14]. Uptake has been rapid in hospitals, but it is only recently that students rounds have begun to be trialled in the UK. In 2016, rounds were piloted for a large cohort of fifth and sixth year medical students using an interdisciplinary panel. The results were promising, with students praising the rounds ability to “*normalise emotion”* and *“promote connectedness*” with colleagues [15]. Another pilot study showed attendee preference for SRs over current written reflective practice [16]. Both these pilots used a multi-disciplinary panel and a medical student specific audience, in contrast to the original SR format of an MDT audience. The impact of this variation should not be underestimated, since SRs allocate more time to audience contribution than they do panellists speeches. Further pilot schemes have continued to show the positive student impact of SR style events, whilst also continuing the medical student only audience [17, 18]. So far only one study has attempted to replicate the existing SR MDT structure in a student setting [19]. Here semi-structured interviews were used to assess the impact of fully inter-disciplinary rounds on a range of student groups. Further positive themes emerged, with students finding unity through shared emotion and also valuing the protected reflective space. Students did however feel a resistance to contributing, questioning if a public forum could truly be considered a safe space.

## Aims

Our study had two main aims. Firstly we intended to add further evidence to the debate around implementation of student SRs. Secondly we aimed to examine the impact of varying the range of disciplines in attendance, asking a question that has not yet been answered before—do medical students truly benefit from reflecting with the MDT? To answer this we chose to measure medical student experience across two rounds, with round one adopting a single discipline approach and round two including various non-medical student groups. We used a mixed methodology approach, using quantitative data to make direct comparisons between the two rounds, and qualitative data to gain deeper understanding of the student experience.

## Methods

### Participants and setting

All Queen Mary University of London (QMUL) medical students on placement at the Trust were invited to attend through a group email. Students in their third, fourth and fifth clinical years were invited. Students were also reminded of the rounds at the end of other teaching sessions. Attendance of the rounds was encouraged but not mandatory. Rounds were run at lunchtime and dates were chosen to avoid any conflicting events. For the second round, other students at the Trust were also invited to attend, with invitations organised via their clinical tutors.

Both rounds were held virtually on Microsoft Teams due to the restrictions imposed by the pandemic. The rounds were facilitated by one of the Trusts POCF certified facilitators and lasted one hour. The research team for the study introduced, observed and closed each round, mimicking the co-facilitator role that a clinical lead would take in a POCF SR [20]. Researchers did not however partake in the facilitation of the rounds themselves. Mental health first aiders also attended to offer support and signposting to students where necessary.

The panel for the first round consisted of four QMUL medical students previously placed at the Trust. The second round’s panel consisted of two QMUL medical students and a pharmacy student. The title for the first round was “Is this what I signed up for?”, and the second was “In at the deep end…”. Titles were taken from previous rounds, and selected to cover similar areas of conversation. Panellists and titles were chosen by the researchers. Panellists were given the round title in advance and then asked to prepare their contributions independently. Each panellist was then pre-briefed by a researcher and the Trust’s SR provider to discuss their contributions.

### Data collection and measures

**Fig. 1:**
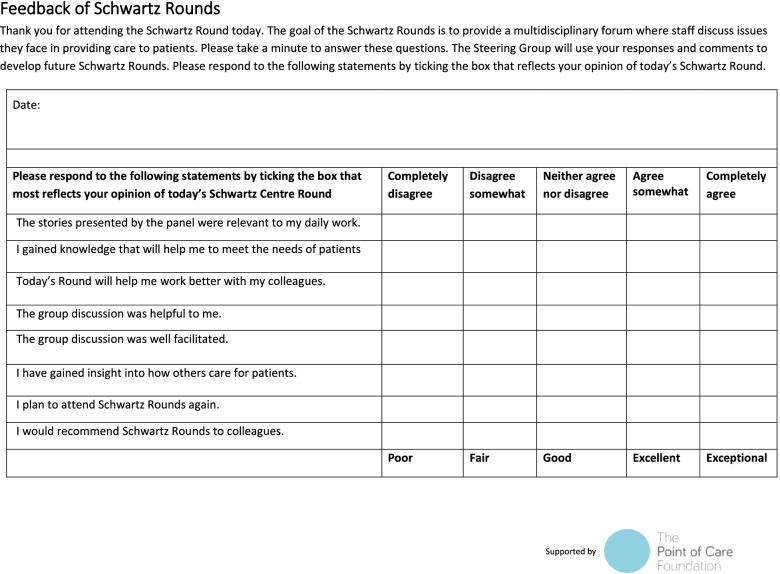
POCF feedback form with statements alphabetized

Following the rounds, all QMUL medical student attendees were invited to complete a standard POCF evaluation form (Fig. 1). This form asked respondents to rate eight statements relating to the value of attending the round, using a 5-point Likert scale. The scale ranged from completely disagree to completely agree, and also included free text space. In order to compare students’ responses the forms were pseudo-anonymised before being analysed. After each round, medical students were invited to join a focus group to discuss their experience, answering pre-prepared questions relating to the scope of the research. Focus groups were facilitated by the study researchers, who undertook an Open University course on focus group facilitation in preparation. Focus group audio was transcribed using a combination of Otter™ software and hand transcription. Focus groups were chosen as the research tool for two reasons. Firstly, they allowed researchers to include a larger number of attendees than would have been possible with individual interviews. Secondly, they provide attendees the freedom to explain their answers, as well as respond and expand on other attendees points.

### Analysis

In order to statistically analyse the two rounds, the quantitative data was converted to a five-point score, where one equals “completely disagree” and five equals “completely agree”. Median averages were then calculated from all those attending. Additional analysis was undertaken on the scores for those medical students attending both rounds. This observed the change in their response to round one and two, to show if the second round had elicited a more positive or negative response to each statement.

**Fig. 2:**
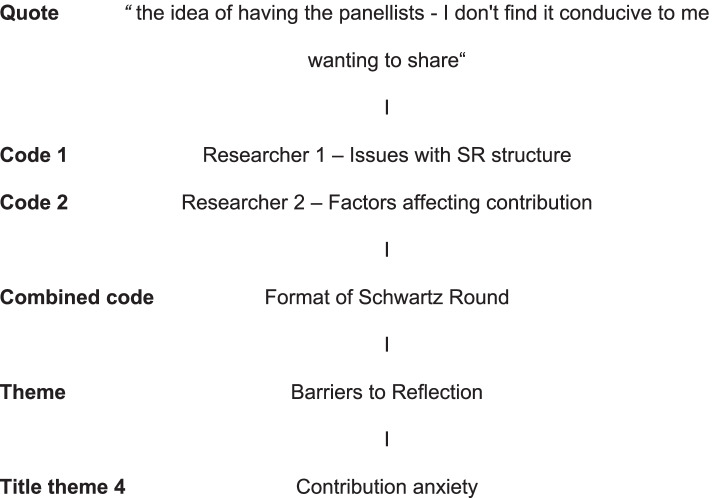
.

Qualitative data from the focus group underwent staged thematic analysis. First, two researchers independently converted the data into codes. Both sets of codes were then combined and inputted into NVIVO™ 12 for further analysis and formation of themes. Finally the themes were grouped into broader title themes. Analysis was modelled on existing stepwise guidance on applied thematic analysis [21, 22], and was structured to follow a positivist exploratory approach. An example of the analysis can be seen below Fig. 2.

## Results

### Attendance and feedback

**Fig. 3:**
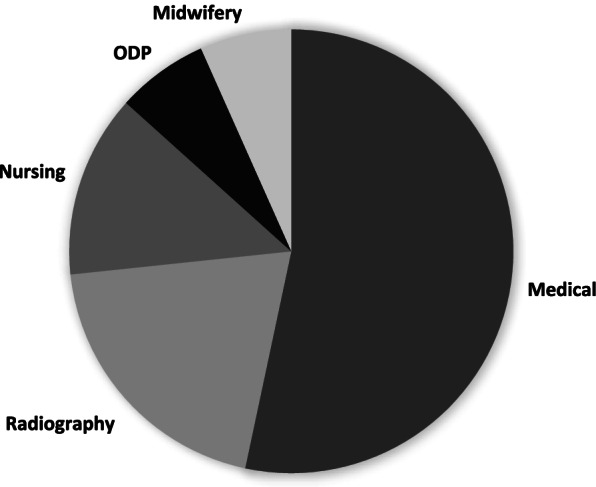
Audience makeup for Multi-disciplinary round

Both rounds attracted similar audience size. A total of nineteen medical students attended the first round, which represented 67.8% of all those invited to the round. Twelve were female, and seven male. Round two had an audience of eight medical students, seven of whom were present for round 1. In addition to this were students from radiography (3), nursing (2), midwifery (1) and operating department practitioners (ODP) (1) (Fig. 3). The gender demographic for round two was six male and nine female. Attendance figures are shown in Table 1.

**Table 1:** Attendance figures for SR audiences and focus groups

Attendees	Round 1	Focus group 1	Round 2	Focus group 2
Medical student	19	10	8	5
Non-medical student	0	0	7	0

Feedback forms were received from 94.7% (18/19) of medical students attending the first round, and 100% (8/8) of the second. The first round was followed by two focus groups of five students each, running in parallel. The second round was followed by one focus group of five medical students. All of those attending the second focus group had been present for the first.

### Quantitative results

**Fig. 4:**
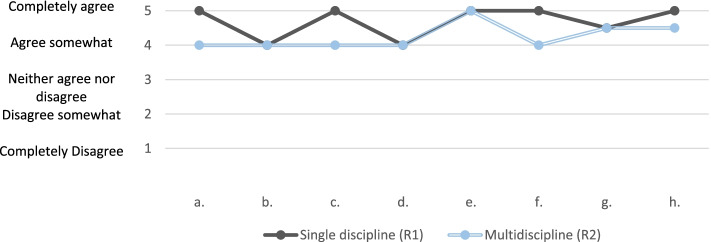
Graph of median responses for both rounds

Median averages were calculated from all attendees and can be seen in Table 2. A comparison of median averages can be seen in Fig. 4. This shows an overall agreement with each statement, with round one eliciting an equal or stronger agreement in all cases. Statements are alphabetised in the order they appear on the POCF feedback sheet on Fig. 1. The median rating for both rounds was “excellent”.

**Table 2:** Median averages for both rounds and net change in response

Question	Median R1	Median R2	Change in median averages	Net change in paired responses
a. The stories presented by the panel were relevant to my daily work	5	4	↓	↓
b. I gained knowledge that will help me to meet the needs of patients	4	4	↔	↓
c. Today’s Round will help me work better with my colleagues	5	4	↓	↓
d. The group discussion was helpful to me	4	4	↔	↓
e. The group discussion was well facilitated	5	5	↔	↓
f. I have gained insight into how others care for patients	5	4	↓	↔
g. I plan to attend Schwartz Rounds again	4.5	4.5	↔	↔
h. I would recommend Schwartz Rounds to colleagues	5	4.5	↓	↓

The responses of the seven students attending both rounds were analysed further. Each students answer was compared for round one and two, to observe if their score changed between rounds. This change can be seen in the final column of Table 2. This column shows that the net change for all statements was either negative or neutral.

### Qualitative results

Thematic analysis produced four overarching themes, which were further subdivided into subheadings.

### Theme 1: concerns with the current reflective curriculum

#### Rigidity of current requirements

There was a dissatisfaction with the current medical school requirements to complete a minimum number of written reflective pieces. This was paired with frustration about being asked to reflect on demand. Also noted was the positive impact the rounds had on these concerns.

*“Reflection for the sake of it is not useful”*

*“Forcing me to write [reflections]… has turned me implicitly against projects like this, but actually, when you participate in them, you realize that there is value to reflection”*

For some, these frustrations had led to a negative attitude towards reflection in general, with students disconnecting from the process.

*“Up until now I’ve just done minimal effort”*

*“I don’t personally reflect unless someone makes me”*

#### Need for flexibility

There was also a desire for more flexibility to be allowed within the reflective curriculum. Views here were more nuanced, with acknowledgement that the form of reflection is dependent on the situation.

*“I think both Schwartz rounds and written reflections both have a place, I don't think it's a case for one or the other is better”*

*“Some more personal things that you do need to reflect about, get with yourself sometimes and sit down and write about them”*

One student did show a preference for group based reflection.

*“I find it would be much better formatted in a way that we have an active discussion”*

The general desire however was for more variation in their reflective options.

*“I think *encouraging* multiple forms of reflection, spontaneous and kind of expected, would be much better.”*

#### Benefits of early reflection

There was a general consensus that access to a SR-like forum would have been especially beneficial from the start of medical school. Two perceived benefits were highlighted; increased togetherness and the normalisation of self-doubt.

*“I think if we had something like this from day one medical school as well, I think it would make people feel much less alienated and not as guilty about having negative feelings while in medical school.”*

Similar thoughts are mirrored by a second student, who acknowledged the empowering effects that group discussion can have.

*“If from day one of medical school I had an outlet for sharing how I felt about things, it would not only bring everyone closer together, but it will make you feel much less guilty and much more empowered about the way that you fail, and that it's okay, and you feel validated and won’t just feel so on your own”*

Early implementation also was seen as a way to mitigate the pressures of medical school and potentially improve mental health.

*“Having SR or similar from day one of med school it could really help a lot with like medical school culture, like competitiveness, and just the whole sense that we're always meant to be on top of everything and know where we were going in life and things like that. And I feel like it could really be hugely beneficial for everyone's kind of mental health throughout medical school”*

### Theme 2: current exposure to the multi-disciplinary team

#### Lack of understanding of other disciplines

Throughout the second focus groups there were repeated references to the separated nature of medical school and other healthcare trainees. This was seen as a source of frustration.

*“As medical students … we don't tend to hear these sorts of reflections from other professions who we are meant to be in a team”*

*“Everyone's still going to be part of the same team at the end of the day but throughout our training we [doctors and nurses] are kept so separate, which seems very strange… it seems like there's a huge disconnect now. And it's a shame because I think we do we do lose a lot of perspective from that.”*

This separation had left one participant feeling their lack of exposure prevented them from understanding the roles of other students.

*“I'm curious about the lives of nursing students and pharmacy students. I don't know what it's like at all. I have no point of comparison.”*

For another student, their limited experiences within the MDT had created a false perception of the role of another healthcare profession. Here, the SR was able to provide a more realistic understanding.

*“All I’ve seen from a lot of pharmacists is sat in MDT room on their laptop. So I feel like it was really good to see and hear beyond … what I'm under the impression of”*

The presence of the MDT was seen not only as a means to improve current understanding, but to prepare for the transition from student to worker. On the topic of integrating with other student groups, one attendee stated.

*“I think it would then really help in the future. When these are our colleagues and we work together as a team to know how we've all kind of studied and how we've all learnt, I think, would be really beneficial.*

#### Improving communication and culture

One student saw the early integration of SRs as having the potential for improving workplace communication.

*“I think [SRs] could really benefit the communication issues between specialties as well, which was a topic that kind of arose in that discussion”*

The same students also noted how SRs had the potential to positively impact on the workplace culture of the NHS.

*“You would reduce the hierarchy, and in the end, improve the mutual respect between people when they talk to each other because there's no need to ever belittle anyone in a workplace. And it's not conducive to anything productive”*

### Theme 3: normalising negativity and moving forward

#### Reassurance through sharing

A unifying theme in both rounds was the reassurance students gained from hearing peers express similar thoughts to themselves. Participants found particular reassurance in more negative feelings like self-doubt and anxiety about the future.

*“It does take a lot to kind of open up and admit that sometimes you do have those thoughts [career doubts]. But for me, it was very reassuring to also know how normal that is.”*

*“Having those moments where you feel like am I really cut out for this? Is this really what I want to do day in day out for the rest of my life? … I found it very reassuring [hearing others]”*

#### Hope for change

Reflections in the round often had a negative perspective, but it was generally seen as a positive that these thoughts and emotions were being shared. This was in part due to the belief that acknowledgment of concerns now was a step towards future change.

*“To see so many people go “ I don't enjoy this, I want to see it different”—Is it a really positive thing? I think yes”*

*“I don't know what any medical student now who doesn't feel the same way the people in this group feel personally. So that gives me hope because we are the future of the hospital force within the medical teams”*

One student saw the collectivization of negativity as a form of progressive critical thinking.

*“I think if you sit and you're critical and you reflect on what is a problem, then you're much more likely to bring about change in that sphere”*

### Theme 4: contribution anxiety

#### Fear of judgement

Whilst the overall impression from both rounds was positive, concerns about various aspects of the SR structure were raised. Some of this stemmed from a fear of judgement from the group.

*“There is that sort of feeling will you get judged, what are other people thinking about your experiences?”*

*“I can at times really be quite negative about medicine. And I feel in the group like that it’s quite hard to put that across without being worried about getting judged for it”*

The focus group for the second round found these contribution anxieties exacerbated by the makeup of the multi-disciplinary audience.

*“I think going into a room with so many people that I've never met before and talking about these things very, very difficult”*

#### Panel and response structure

Further concern was raised regarding the format of the SR, wherein a panel will prepare their speech in advance but the audience responses are always spontaneous.

*“I think it actually sets you up to feel self-conscious about not having a particularly good structure to what you're saying”*

*“[on speaking after a panellist] whatever I say is going to sound crap now, because I'm just going to ramble, it's not going to sound like this really well prepared”*

#### Group size

Anxieties were magnified by the idea of public speaking in front of a large group, something that was raised repeatedly in both focus groups.

*“I think there’s quite a few people in there which can be quite intimidating to be raw and emotional”*

*“I'm not sure I would be able to articulate what I want to say … And be able to admit those sorts of things in such a big group with so many people”*

#### Online platform

Traditionally rounds are run face to face, but the pandemic forced these rounds to be held virtually over Microsoft Teams. Repeated criticism was aimed at the virtual platforms limitations.

*“Especially online, if you can't see people's faces, it's really disconcerting, kind of spilling your heart out’*

*“I think definitely it being on Teams and not being able to see everyone will take away the personal aspect of it where you might feel more comfortable to share”*

## Discussion

### Attendance

Given the small student cohort available for the study (*n* = 29) and the voluntary nature of the rounds, attendance for the first round (*n* = 18) at was surprisingly high. A significant drop-off occurred for the second round (*n *= 11), although the presence of other student groups meant student audience numbers for both rounds were similar (*n* = 19, *n* = 16). A possible explanation for the drop-off is the consenting procedure. This was done face to face in the week preceding the first round, thus also acting as a reminder to the students. Also relevant may be the timing of the second round, which occurred 12 days after round one, as opposed to the month gap that the POCF recommend [6]. Drop-off may also have been due to prioritising self-study over attendance, since for many students this was their final placement before examinations. The high feedback scores from the first round suggest drop-off was not due to dissatisfaction with the experience. One might also expect the students that attend both rounds to be those most engaged with SRs as a concept, or those that benefited most from the first round. The data in fact suggests otherwise since the scores of double attenders were mostly lower, although the MDT presence may account for that.

### Quantitative data

Our study used quantitative data for two purposes; to evaluate the success of each round, and to compare student experience between the two. The independent variable in this comparison was the presence of multiple student disciplines. This study is the first to attempt a direct comparison in this way.

The median averages showed uniform agreement with each POCF positive statement. The statements can be seen in Fig. 1 and cover relevance, benefit to attendee, and future re-attendance or recommendation. This general evidence towards the success of the rounds is backed up by the overall median rating for each round, which was “excellent” for both. The statement that elicited the lowest scoring response in both rounds was “I gained knowledge that will help me to meet the needs of patients”. This may reflect the content the panellists and audiences raised, which has been kept confidential. Since audience participation is spontaneous, improving this rating may be achieved by more clinically focussed panels. This would need to be balanced against the aims of SR, which are not run to be a clinical teaching session.

The lower scores in the MDT round may be linked to the contribution anxiety elicited by the presence of strangers in the meeting. Reflection is a deeply personal process, and medical students are very much in their infancy to the concept. This argument is discussed later in the thematic analysis. We must also consider if the short gap between rounds diluted the impact of the second round. Further to this is the familiarity of students to the concept of SRs. Only one student from the paired group had experienced a SR before attending round one, which of course cannot be said by round two. This variation creates potential for response bias.

### Thematic analysis

This study used thematic analysis to explore student understanding and reaction to SRs, producing four major themes.

### Concerns with the current reflective curriculum

There was general frustration from the group at the rigidity of mandated written reflection, which for some had resulted in disengagement in the process. Motivating students to reflect is a challenge [23], and the subjective nature of motivation makes consensus difficult to find. An international focus group study by Sargeant et al. [24] consistently found student engagement to correlate with the quality of feedback they received, be it from supervisors or peers. Further understanding of reflective motivation is found in a 2007 study by Driessen et al. which examined medical student portfolios [25]. The results suggested that understanding the reasoning for reflection is a more important predictor of engagement than the method of reflection itself. Taking this into account, the students’ frustrations here may represent two issues: a dislike of the inflexibility of written form and secondly an issue with the medical school’s communication of its own reflective curriculum. The findings here also tie in directly with the results of a previous SR pilot study, which found a strong preference for SRs over written reflection [15]. Written reflection in general fails to engage the full range of student learning styles, ignoring both the pragmatic and the active learner [26]. The requirement for a set number of separate essays also fails to allow for a reflective cycle, instead creating several separate linear journeys. This contradicts the seminal theory developed by Kolb [27] which finds reflection to be a process that must progress cyclically and logically. The GMC document *The Reflective Practitioner* [13] explains reflection as a subjective variable process, openly drawing on the established theories of Honey Mumford and Kolb [26, 27]. If medical schools intend to improve student reflective engagement then they too must consider how their future assignments can incorporate the knowledge from these established theories. This begins by appealing to all types of learner, as well as encouraging individuals to re-assess and re-validate their own development. It also requires students to have the option to reflect in a way that they themselves find most effective, be it a SR or otherwise. Perhaps the answer is for those setting the assignments to show greater flexibility and creativity. A successful example of this comes from Brown University in the USA, where surgical students were asked to submit “tweet” style (140 characters) reflections throughout their rotation, resulting in more impactful and actionable reflections [28].

### Current exposure to the multi-disciplinary team

Consensus was uniform that greater integration of the MDT is needed at medical school. MDT presence in these rounds provided students with insight and appreciation of other healthcare roles, and was also seen as a potential tool for improving inter-speciality communication within the NHS. This is an especially important finding when considering the NHS long term plan, which sees collaborative working as a central tenet of its redesign [29]. The fact that students saw MDT integration as a route towards a culture shift is particularly important for an institute like the NHS, which is no stranger to the concepts of bullying and harassment [30]. There was also consensus on the need for earlier integration of the MDT into medical school. This was a more general point about current curriculum inadequacies, rather than advocating specifically for SRs as the solution, although student rounds are one means of providing early integration.

### Normalising negativity and moving forward

The third theme of normalising negativity and self-doubt builds directly on similar findings from previous student pilots [15, 17]. Our participants expand on these themes, seeing them as potential routes to future change. This aligns with the GMC document *The Reflective Practitioner,* which proposes that the clinician “thinks analytically… using the lessons learned to maintain good practice or make improvements” [13]. *The Reflective Practitioner* goes on to recommend group reflections as a mechanism to enact complex systemic change. It is interesting that some participants already knew one another from previous placements, but this was the first time they had heard their peers self-doubts. This is perhaps indicative of the unique space for sharing that SRs can provide. The critical appraisal of these shared misgivings was also seen as a potential mechanism for systemic change. Further exploration of this idea may be limited by one of the POCF’s core principles—rounds exist to share but not solve problems [6].

### Contribution anxiety

The size of the group was highlighted as one cause for these anxieties. Only one previous pilot had comparable numbers (*n* = 28 to 45), and again anxieties over group size was a major theme [15]. This highlights the first paradox of the SR, that it exists in part to reduce attendee stress, but to do this it relies upon public speaking which is in itself a common stressor. Participants acknowledged that the SRs attempted to offer a safe space, but despite this still raised concerns about being judged by their peers. Concerns were more numerous in round two, commenting specifically on the discomfort of sharing with strangers. It’s worth noting that these concerns are being voiced for rounds with relatively minimal total attendance numbers (*n* = 33, 32). The Point of Care foundation recommends a minimum audience of ten people, but no upper limits [6]. This represents the second paradox of the SR: by being open to all, audience numbers have the potential to spiral, which may then negatively impact the success of the round. The online platform likely exacerbated problems here, since Microsoft Teams only showed the videos of the panellists, facilitator and speaker, but in physical SRs speakers are likely to recognise their colleagues in the crowd. There is no easy answer here, since showing all attendees on Teams will likely exacerbate the concerns over group size, and would also be difficult on a small screen with variable WIFI.

### Qualitative vs Quantitative results

There are contrasting findings between the quantitative and qualitative results. The focus groups showed clear advocacy for MDT integration, but feedback forms showed a negative correlation when the MDT was present. Quantitative data in this study has low statistical power, and conclusions from it are drawn with far less certainty than the qualitative findings. It should also be noted that the feedback scores do show an overall advocacy for SRs, irrelevant of the MDT presence. Interestingly the focus groups were advocating strongly for the early integration of the MDT and for more SRs, but only one student spoke specifically about the benefit of the MDT in the round they had experienced. It is therefore possible that different reflective formats would be better placed to introduce the MDT.

The focus groups were able to examine attitudes to reflection and SRs as concepts, whereas the quantitative data is more dependent on the content of the rounds – panel stories, audience comments, themes of round. Without similar studies for comparison, it is difficult to say definitively if the quantitative outcomes were due to the content or format of the rounds.

## Recommendations

- There is good evidence to recommend running further student SRs, in both single and multiple disciplinary format.
- Medical schools should consider how they can include earlier integration of the MDT into medical school training, as well as showing greater flexibility and theory in their reflective requirements.
- It may be most beneficial to use SRs as a space for medical student reflection, and introduce the MDT in other areas. Given that this is the first study to trial student disciplinary variation, and the limitations of the quantitative data itself, this area should be researched further before any permanent changes are made to the SR audience.
- Finally, to ensure maximum audience contribution organisers should keep group size small (we suggest no larger than our pilots) and discourage fully scripted panels.

### Questions unanswered

- Should rounds return to their original physical format, and if they are to stay online then which virtual platform is best suited for them?
- How does the MDT presence impact reflection in other hospital student specialities?
- Will these rounds have any effect on the reflective and clinical practice of the attendees later in their career?
- Does early integration of the MDT have an impact on clinical MDT outcomes?

## Limitations

### Researchers

It is important to be up front about the various ways we as researchers may have influenced this study. We attempted to minimise our involvement throughout but this was not always possible. A degree of selection bias is likely in our choice of panellists. We attempted to run focus groups as neutral facilitators, however it is probable we will have exerted some unintentional control and direction over the discussion. We also attempted to reduce this likelihood by completing an online facilitation course offered by The Open University beforehand [31]. The same concerns exist regarding our completion of thematic analysis, which is a highly subjective process. It is hoped by initially coding the data independently we have reduced some of the subjective risk.

### Data

Likert scales are a well-researched tool for gathering feedback, but they are susceptible to central tendency bias, acquiescence bias and social desirability bias [32]. Any one of these three may explain why less than 3% (6/208) of the total responses were in disagreement with a statement on the feedback form. We must also acknowledge the weak statistical power of a comparative analysis based on a seven person data set, meaning conclusions should be made tentatively at best. This is why our recommendations do not currently advocate for any changes to the makeup of an SR audience. Furthermore, the nature of voluntary attendance means those who choose to attend are likely to be more engaged in the concept from the start, causing a positive skew on feedback.

### Implementation

This project was possible thanks to the generosity of staff within the Trust and university. In practice running rounds on a regular basis takes time and significant funding, and requires facilitators to undergo formal training days through the POCF. NHS staff who are currently trained in running SRs are likely to already be fully occupied delivering Trust wide rounds. The addition of student rounds would therefore require further investment in three resources the NHS already struggles with; time, money and staffing. Whether the monetary resources would come from the university or the hosting Trust is also unclear.

## Conclusions

SRs are increasingly being seen as a means of providing medical students with an alternative form of reflection. This study adds further evidence that student SRs can benefit workplace communication and reflective engagement. There is also evidence that students would benefit from earlier integration of the MDT into their course. The data in this study tentatively suggests that an SR is not the ideal setting for this integration, as it may be detrimental to their overall success. Further research is needed into this specific area before any structural changes to student rounds are considered. Any future changes to the rounds should attempt to mitigate contribution anxiety by maintaining small attendance numbers and fostering a supportive space.

## Data Availability

The datasets used during this study are available from the corresponding author on reasonable request.

## Abbreviations

GMC: General Medical Council
MDT: Multi-Disciplinary team
NHS: National Health Service
NIHR: National Institute for Health and Research
POCF: Point of Care Foundation
PAH: Princess Alexandra Hospital
QMERC: Queen Mary Ethical Research Committee
QMUL: Queen Mary University of London
SR: Schwartz Round
